# Typology of Tomato Cropping Systems and Determinants of Preharvest Losses in Western Cameroon

**DOI:** 10.1155/2024/5625648

**Published:** 2024-11-01

**Authors:** Roland Wilfried Titti, Anne Stéphanie Etoga, Pierre Germain Ntsoli, Georges Marius Kossi Etame, Asafor Henry Chotangui, Réné Mbonomo Bikomo, Aoudou Yaouba

**Affiliations:** ^1^Department of Agriculture, Faculty of Agronomy and Agricultural Sciences, University of Dschang, Dschang, Cameroon; ^2^Department of Rural Socio-Economy and Agricultural Extension, Faculty of Agronomy and Agricultural Sciences, University of Dschang, Dschang, Cameroon

**Keywords:** cropping systems, preharvest losses, tomato, typology, western Cameroon

## Abstract

Preharvest losses, which are often neglected, limit the availability of fresh tomato fruit to varying degrees in tomato-based cropping systems in Cameroon. Increasing tomato yields requires identifying, understanding, and controlling the factors responsible for preharvest losses in the identified cropping systems. Field surveys were conducted in three production areas of western Cameroon (*Foumbot*, *Bansoa*, and *Dschang*) to characterize cropping systems and growers, quantify production losses, and identify causal factors associated with losses and proffer solutions. One hundred and ninety-six growers were interviewed in 13 villages, using purposive sampling to select zones and simple random sampling to select growers. Factor analysis for mixed data (FAMD) and canonical discriminant analysis, combined with multinomial logistic regression, were used to analyze the collected data. The FAMD results indicated that 72% of the farm variability was expressed through technical route, preharvest losses, and technical mastery. The tomato-based cropping systems were classified into three types: (1) a pure cultivation system that consumes excessive synthetic fertilizers and results in significant losses; (2) a pure cultivation system that consumes high amounts of synthetic fertilizers and moderate amounts of organic fertilizers with moderate losses; and (3) a cultivation system that involves moderate synthetic fertilizer consumption, extremely low organic amendment, and low losses. Preharvest losses can be significantly affected by the unregulated use of synthetic fertilizers. The study's implications are many, affecting agricultural practices, policy, economic stability, and food security in Cameroon. A more sustainable and productive tomato industry can be achieved by addressing preharvest losses through informed strategies. To address this issue, it is crucial to establish fertilization protocols that consider the soil's fertility status and the tomato's essential macro- and micronutrient requirements.

## 1. Introduction

Agriculture contributes strongly to the socioeconomic development of populations as it employs more than 52% of the active population in Africa [1, 2]. Market gardening influences humans through its status as the most productive system in Africa [2, 3]. This sector offers huge export potential for fresh produce, in addition to its use in most programs aimed at reducing human nutrition problems [2, 4]. In most African countries, vegetables such as tomatoes, cabbage, and French beans are high-value crops [5].

Tomatoes (*Solanum lycopersicum* L.) are a common vegetable crop in Cameroon, with 329,000 growers engaged in its cultivation. The majority of these growers are engaged in family farming, which is characterized by small cultivated areas and artisanal agriculture [4]. The crop is primarily grown in the West region, representing 62% of the national production, and generates direct and indirect jobs for Cameroonians [4]. It accounts for about 35% of the combined amount of fruit and vegetable consumption [4, 6].

However, the average yield obtained in Cameroon (12.6 t·ha^−1^) is still far from that obtained in Egypt (41.6 t·ha^−1^), Tunisia (57.7 t·ha^−1^), and Morocco (94.5 t·ha^−1^), which remains the leading producers in Africa [7]. Pest pressure has been identified as a significant constraint due to crop losses inflicted on market gardeners [8, 9].

Beyond the health aspect, soil fertility management is increasingly one of the constraints to the intensification of market gardening in western Cameroon [10]. This situation directly influences the behavior of farmers in this part of the country, who rely on the misuse of synthetic fertilizers [11]. However, their ability to rapidly provide the mineral elements needed for crop growth and development does not overshadow the persistent problem of limited production [4]. Reference [12] pointed to steadily declining fertilizer use efficiency in recent decades. One consequence of poor fertilizer management is nutrient imbalance, which can significantly affect flower and fruit drop, limiting production [13–16]. Similarly, the intrinsic quality of the fruit and its shelf life would be affected by the fertilization practices of the crop [17]. Hence, the reservations expressed about the uncontrolled use of synthetic fertilizers [12], not to mention their impact on the environment and the consumer [18, 19].

In the West Region, the most important supply chains are those of *Foumbot* (*Mangoum*, *Fosset*, *Baïgom*, and so on), which account for 525,760 tons per year, and *Dschang* (*Balessing*, *Bansoa*, *Dschang*, and so on), which account for 50,640 tons per year [4]. These supply chains alone create about 750,000 jobs, contribute on average 37.5% to the total income of farmers, and play an essential role in achieving food security in the country [3, 4]. However, the negative impact of preharvest losses (PHLs) during the production phase is highlighted in the assessment of these production chains. These losses are much more quantitative than qualitative during the preharvest period and are mainly reflected in the loss and decay of unripe and ripe fruit, which may be caused by pathogens, pests, pedoclimatic factors, inputs, and technical management [2, 4, 20].

In addition, estimates of food losses indicate that 30%–50% of food production is lost between the field and the consumer. In the western Cameroon region, the level of fresh tomato losses was estimated at between 27% and 31.7% during the preharvest period [4]. Reducing the loss of edible produce is therefore becoming a critical challenge in eradicating hunger in the world, especially as the global population growth observed increases the pressure on food [4, 21]. Likewise, these losses also correspond to an unnecessary use of essential resources (water, fossil energy, and minerals) in the delicate context of their scarcity and the need to reduce the environmental impact of agriculture [22–25].

Previous research on tomato production in this part of the country has largely ignored cropping systems and PHLs, focusing instead on the effects of disease and fertilization on yield. It is essential to identify the drivers for action that would make it possible to limit production losses. Farm typologies are increasingly used for their ability to identify similarities and differences within farms, as well as constraints related to the diversity of cropping systems observed [26, 27]. This is fundamental for the formulation of interventions adapted to the local production context. The objective of this work was to identify the typologies of tomato-based cropping systems, as well as the factors associated with PHLs. The remainder of the study is organized as follows.  Section 2 presents the conceptual and empirical framework of the study.  Section 3 presents the results, and Section 4 presents the discussion.  Section 5 concludes the study.

## 2. Materials and Methods

### 2.1. Location of Study Area

The study was carried out in the localities of *Foumbot*, *Bansoa*, and *Dschang*, located in the Type III agroecological zone (Western Highlands) of Cameroon. The climate is equatorial and comprises two zones: (1) an equatorial zone of the Guinean type, characterized by annual rainfall of more than 1500 mm, an average temperature oscillating around 25°C and an amplitude of 2°C; (2) an equatorial zone of the Cameroonian type, characterized by a unimodal rainfall regime (2000–11,000 mm annually) and average temperatures of 22°C [28, 29].

There are generally two types of soils in the study area: (1) acidic ferruginous soils, clayey red or yellow, with limited ability to retain minerals, and (2) extremely fertile nitisols, rocky sediments from volcanic slopes [29]. The vegetation is mainly characterized by Guinean–Sudanian savannahs at very degraded altitudes, semideciduous forests, and montane forests [29, 30].

### 2.2. Methodology

Field surveys were conducted with tomato growers in February 2021 for *Foumbot* and in March and August 2022 for *Bansoa* and *Dschang*, respectively. These surveys consisted of personal interviews using a semistructured questionnaire designed to obtain the sociodemographic characteristics of producers and the technoeconomic characteristics of agricultural holdings (Table 1). The data were collected using *KoboToolbox*, a database collection and management application. The design and validation of the questionnaire was done in several steps: (1) literature review to identify the key variables to be studied, (2) consultation with experts and growers to validate the variables, (3) developing the first draft of the questionnaire, (4) pilot testing the questionnaire with a small sample of growers, and (5) revising and finalizing the questionnaire based on the results of the pilot testing.

### 2.3. Study Population, Sample Size, and Sampling Method

The studied population consists of tomato growers in the localities of *Foumbot*, *Bansoa*, and *Dschang*. The sample size was determined on the basis of the percentage of agricultural households producing tomatoes, using Dagnelie's equation (1) for a population of unknown size [31]:

(1) $$\begin{array}{c} n=\frac{Z\alpha^{2}\times P\left(1-P\right)}{d^{2}}, \end{array}$$

where **n** = sample size; **Z** = critical value of the standard distribution at the required confidence level; **P** = proportion of households producing tomatoes; and **d** = maximum tolerable error, 1% ≤ *d* ≤ 15%. For this study, *α* = 5%, Z*α* = 1.96, and *d* = 9.8%.

The towns and villages that were selected for the study were chosen on the basis of a purposive sampling technique [27]. The criteria that were used to justify their selection were the production capacity and the existence of a distribution chain with a significant flow at the national level. *Foumbot* is the country's largest tomato production basin and supply chain, with an annual production of around 525,760 tons, supplying the country's major cities and even several countries in the Central African subregion (*Gabon*, *Guinea*, and *Central African Republic*). *Dschang*, with its villages (*Balessing*, *Bansoa*, *Bafou*, *Dschang*, etc.), is the second largest supply chain in the country, with an annual production of about 50,640 tons. Its distribution chain is much more focused on the main consumption centers of the country (*Douala*, *Yaoundé*, and *Bafoussam*). This status, given their importance in both the production and distribution of fresh food, justifies the selection of these localities as study areas.

The proportion of tomato-producing households in each locality was obtained after presurveying 150 households, based on one respondent per household. At *Foumbot*, five villages were presurveyed, giving *p* = 0.185 and *n* = 60 producers. In *Bansoa*, *n* was 69 farmers, for a *p* of 0.221. The same exercise in *Dschang* allowed us to determine that *p* = 0.215 and to calculate *n* = 67 farmers. Using simple random sampling [26, 27, 32], a total of 196 growers were surveyed.

### 2.4. Data Analysis

The data collected were subjected to multivariate and descriptive analyses (frequency, mean, and standard deviation), which were used to summarize and understand them. The data on the sociodemographic characteristics of the tomato growers and the characteristics of the farms were subjected to the chi-square test and Fisher's exact test [27, 33]. The classification of cropping systems in the study area on the basis of the survey data was done in two steps: (1) a factor analysis for mixed data (FAMD) was performed to obtain an intermediate representation of the data [27, 32, 33], and (2) then, a hierarchical cluster analysis (HCA) was performed on the basis of the factors that represented the FAMD [27, 33]. In order to identify the discriminant variables of the different clusters that were obtained, a canonical discriminant analysis (CDA) was carried out [33]. Finally, a multinomial logistic regression (MLR) was performed on the data collected to identify the determinants of losses during the preharvest phase [34, 35]. The chosen analytical method combines six complementary approaches to study tomato-based cropping systems and the determinants of PHLs in western Cameroon. FAMD and HCA identify latent structures and typologies of cropping systems, while CDA and MLR help identify the determinants of PHLs and predict the probability of losses. Descriptive analysis, chi-square test, and Fisher's exact test complete the approach by describing cropping system and PHL characteristics and testing associations between variables. The combination of these approaches allows for a comprehensive understanding of cropping systems and the factors determining PHLs [27, 32, 33, 35]. The analyses were performed with R-4.3.1 software.

## 3. Results

### 3.1. Typology of Tomato Cropping Systems in Western Cameroon

**Figure 1:**
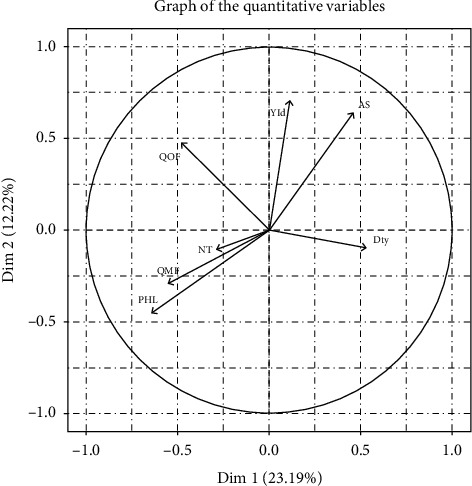
Graph of quantitative variables that characterize tomato growers in western Cameroon.

**Figure 2:**
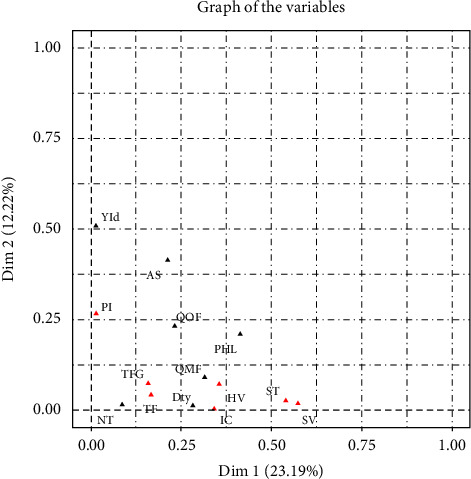
Graph of the variables that characterize tomato growers in western Cameroon.

The results of the FAMD capture 72.3% of the variability among tomato growers. Fertilization and PHLs remain the first factor of diversity among tomato growers in the Western Highlands of Cameroon, explaining 23.2% of the observed variability (Table 2). Moreover, the area exploited and the technical performance constitute the second factor of variability, capturing 12.2% of the variability. In addition, nursery duration and seeding density represent factors that explain 6.1% and 5.8%, respectively, of the variability. PHLs, the amount of organic manure and synthetic fertilizers applied, and the seeding density have a negative impact on growers' performance (Figure 1). These results underscore the critical importance of optimal fertilization and reduction of PHLs in improving the productivity and profitability of tomato production systems in western Cameroon. Further research will be required to identify the best management approaches for these conditions, while the area and duration of the nursery positively influence the performance of tomato growers. The positive relationship between area, nursery duration, and grower performance underscores the importance of optimizing these factors to improve the productivity, economic viability, and sustainability of tomato production systems in western Cameroon (Figure 2). This finding can guide agricultural policies and support programs aimed at improving tomato production in the region.

**Figure 3:**
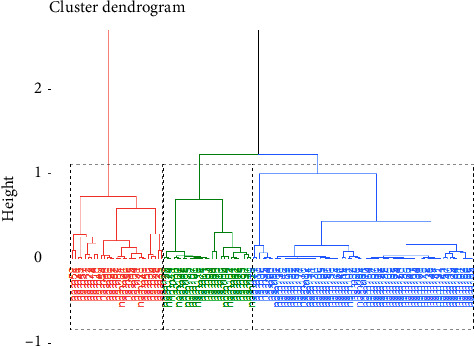
Hierarchical cluster of tomato cropping systems.

**Figure 4:**
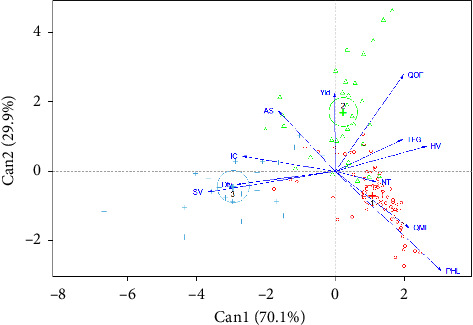
Position of tomato cropping systems on the first and second factors (Dimensions 1 and 2) derived from canonical analysis.

The HCA based on FAMD has made it possible to build three distinct cropping systems: Type 1, Type 2, and Type 3 (Figure 3). The variables (qualitative and quantitative) describing each type are represented in Table 3. CDA revealed that the first two canonical axes were significant (*p* < 0.05) with 70.1% for the first axis and 29.1% for the second (Figure 4). These two canonical axes suffice to identify the variables that distinguish the tomato grower cropping system. The plowing (Pl) and PHLs were significantly correlated with the first canonical axis (Table 4). The second canonical axes were significantly correlated with density (Dty), PHL, quantity of organic fertilizer (QOF), intercropping (IC), and the use of standard varieties (SVs) and hybrid varieties (HVs) (Table 4). These seven variables are the most discriminant of the three tomato cropping systems. Type 1 represents pure cropping system with elevated levels of synthetic input and high PHLs; Type 2 represents pure cropping system with medium levels of synthetic input and moderate PHLs; and Type 3 represents associated cropping systems with medium level of synthetic input and very low PHLs (Tables 4 and 5).

### 3.2. Determinants of Preharvest Tomato Production Losses

**Figure 5:**
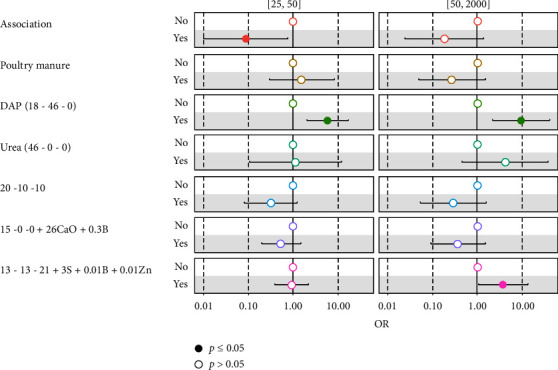
Determinants of tomato preharvest loss in Western Highlands of Cameroon.

PHLs recorded by growers in tomato-based production systems are significantly (*p* < 0.05) associated with cultural practices, the use of diammonium phosphate (DAP), and 13-13-21. Thus, growers carrying out the crop association recorded the lowest market losses (less than 25 baskets). The use of the DAP results to 5.83 and 9.31 times more PHLs for growers with losses between 25 and 50 baskets and for those with losses of more than 50 baskets. Finally, the use of 13-13-21 fertilizer would lead to 3.67 times more PHLs among growers with losses greater than 50 baskets. This shows that growers do not control the use of synthetic fertilizers and that their fertilization strategy causes nutritional disturbances within the plant (Figure 5).

### 3.3. Sociodemographic Characteristics of Tomato Growers

**Figure 6:**
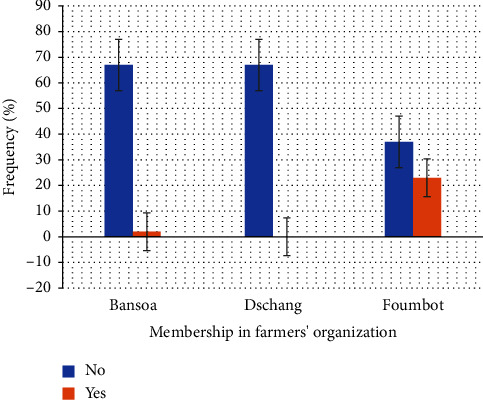
Membership in a tomato growers' organization, depending on location.

**Figure 7:**
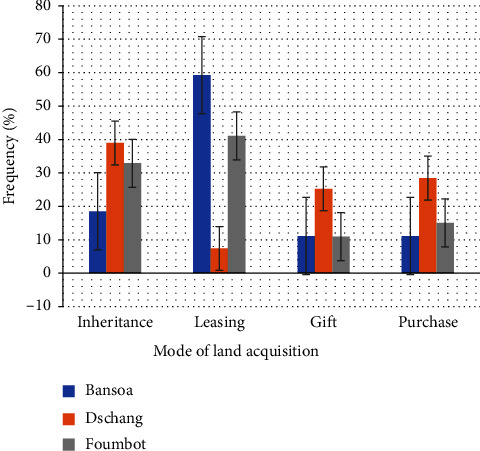
Mode of land acquisition by tomato growers.

**Figure 8:**
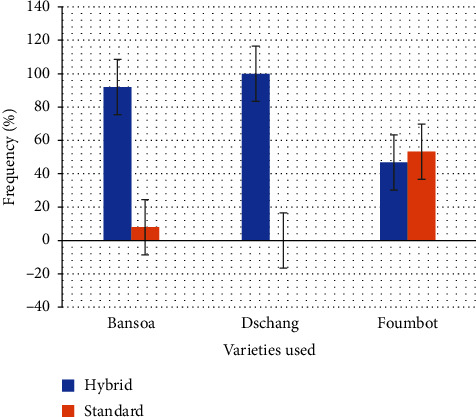
Varietal preferences of tomato growers in western Cameroon.

Tomato production in the locality of *Bansoa* is carried out by an indigenous family with areas of less than 1 ha (Table 6). The involvement of young people, mainly men, in tomato production is strong and indicative of a search for better living conditions, or a more efficient way of funding a family and/or providing for their families (Table 6). The language barrier and the level of education are favorable to the support and development of growers in the use of new tomato production practices. However, this support is limited and difficult to achieve because of the lack of grouping in farmers' organizations (FO) specialized in tomato production (Figure 6). Production practices focused on the conservation of soil resources are unsought and applied by growers in this locality, as demonstrated by the dominant mode of acquisition of arable land, namely, leasing (Figure 7). In addition, it is possible that risk-taking in investments and the choice of techniques are higher for these growers (Figure 8).

In the locality of *Dschang*, tomato production remains the work of the natives through family farms on small plots of land. As in *Foumbot*, production is dominated by adult men (Table 6). The search for securing production income through limited risk-taking is properly expressed by the dominant marital status (Married). Moreover, the conservation of productive resources remains a priority for growers in this locality, as highlighted by land acquisition methods and land pressure in the locality (Figure 7). As in *Bansoa*, the growers of *Dschang* are open to exchange and willing to be accompanied. However, they should still be associated with FO in order to reduce the time and cost to achieve these objectives (Figure 6).

In contrast to *Bansoa* and *Dschang*, tomato growers in *Foumbot* produce on areas of up to 8 ha. Moreover, age seems to reflect a better knowledge of tomato production processes by growers. Providing information and support to growers in the *Foumbot* area is easier, but efforts are still needed to bring growers together in FO (Figure 6).

**Figure 9:**
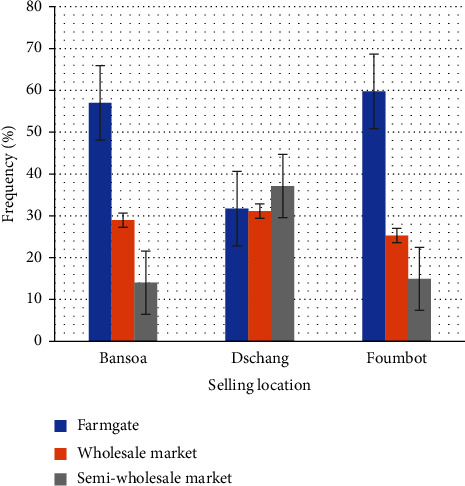
Tomato production sales point.

In addition, regardless of location, growers secure their income by influencing the point of sale. However, growers in *Bansoa* and *Foumbot* are less burdened by transport and prefer to sell in the field. This strategy highlights the lack of financial resources at the end of production. These growers choose to sell to a wholesaler with transport facilities in order to avoid the cost of transport to the point of sale (Figure 9).

### 3.4. Cultivation Technique and Process

Tomato production in the localities of *Bansoa* and *Dschang* is of the conventional type, extended from the point of view of sowing density. It is sown in a simple in-line configuration, with different arrangements between lines and on the line, and is characterized by a lack of recommendations for sowing density in the locality (Table 7). As water conservation remains a key element of out-of-season tomato production, growers adapt their soil preparation methods according to the production area. The ridges are built either to conserve water or to avoid hydromorphic in the shallows (case of *Foumbot* on the very high ridge). Flat plowing is used to promote capillary uplift in unflooded shallow areas (Table 7). In contrast to *Dschang* and *Bansoa*, where tomato growers mainly practice sequential cultivation (tomato, maize and/or beans, cabbage), those of *Foumbot* have developed a rotation system and both use of SV and HV (Figure 8).

**Figure 10:**
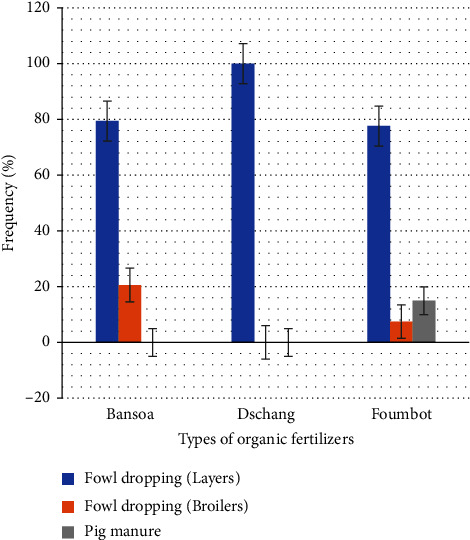
Organic matter preferences of western Cameroonian tomato growers.

The intensification of tomato production in the *Foumbot* locality shows the involvement of a reasonably efficient support system and a significant need for training to improve the productivity of tomato farms. Organo-mineral fertilization is widespread in all localities (Table 8) with a higher preference for layers fowl dropping (Figure 10). However, the fertilization process is poorly known and the importance attached to the organic amendment is relative, or growers consider their soil sufficiently fertile and do not increase the effect of the amendment applied. It is a fertilization based on a multitude of formulations and leads to nutrient imbalances (Table 8).

### 3.5. Disease and Pesticide Managements

In most cases, growers in *Bansoa* show a greater ability to identify disease symptoms, but those in *Dschang* are the most experienced in identifying diseases associated with these symptoms (Table 9). The most conventional mode of chemical disease management is the simultaneous use of systemic and contact molecules in *Dschang*, *Bansoa*, and *Foumbot*. The use of biopesticides remains mixed because the more significant proportion of the use of this alternative to synthetic products, observed in the locality of *Foumbot*, is low. Finally, growers in *Dschang* show a better understanding of plants that can play the role of biological pesticides (Table 9).

### 3.6. Crop Production, Preharvest, and Postharvest Loss

The agricultural practices of *Bansoa* growers result in a high average amount of harvest per season, even if the average yield is reduced, some growers in the locality achieve satisfactory results in their context (Table 10). In the same way, their field management techniques can minimize PHLs and harvest losses, although some growers observe considerable losses depending on the cultivated area. The technical management of the *Foumbot* field is similar to *Bansoa*. There is up until now a positive difference in minimum and maximum yield, and therefore, the mean yield is higher than that observed in *Bansoa*. Growers in *Dschang* produce the lowest yield at harvest, with a relatively low average yield for a far from satisfactory maximum. Their technical skills represent an advantage in stabilizing losses to achieve the lowest maximum losses.

## 4. Discussion

### 4.1. Typology of Cropping Systems

The typological analysis of tomato cropping systems in the Western Highlands of Cameroon shows that there is diversity among cropping systems. This variability is mainly expressed in production losses (preharvest) and organic and synthetic fertilization, which account for 23.18% of this diversity. Three cropping systems were identified: PHL, organic amendment, synthetic fertilizer, cultivated area, nursery life, and density are the main differentiators of these systems. Most of these variables have been identified as playing a key role in diversifying producing systems [26, 27, 32].

The first type is a pure cultivation system that is excessive in synthetic fertilizer consumption and results in significant losses. The excessive use of synthetic fertilizers can be attributed, first of all, to the strong involvement of men who, in their desire to increase their income, choose high-input systems [27, 32]. In addition, lack of knowledge of soil fertility status leads to inappropriate soil fertility management [10, 36]. Indeed, the physicochemical characteristics of the ferruginous soils found mainly in this part of the country are known to have limited availability of nutrients essential for crop growth and development [36–38]. This would somewhat justify the trend toward increased use of synthetic fertilizers, as they can release nutrients into soil in large quantities and short time [39]. This practice would ensure satisfactory production for actors in the sector [11]. However, reservations have been expressed about the involvement of synthetic fertilizers in production losses observed in some crops [12]. Imbalanced nutrient levels in plants can have a major impact on flowering and fruit set, resulting in reduced yield [13, 15]. Although it is true that in some cases these losses are not only due to nutritional factors, but also to physiological and pathological ones. Some diseases can cause up to 100% loss of production before fruit ripening and harvesting by attacking all plant organs [2, 40, 41].

The second type is a pure cultivation system that uses high amounts of synthetic fertilizers and moderate amounts of organic fertilizers with moderate losses. Indeed, the cultural practices (varieties, seeding rows, pure cultivation, and type of fungicide) are similar to those of the first type. A difference can be observed in the type of tillage, which in this case is preferably generalized flat. When done well, this type of tillage allows for good development of the plant's root system and promotes good drainage and aeration. It can therefore have a positive influence on the plant's productive performance [42]. In contrast to the first type, growers in this group use large amounts of organic matter in production. Under certain conditions, organic fertilizers applied in certain quantities can produce as much or more than synthetic fertilizers [38, 43–45]. The ability to improve soil properties becomes a significant grower benefit beyond the residual effects of organic amendments [43, 46, 47].

The third type is a cropping system with moderate synthetic fertilization, extremely low organic amendments and low losses. This characteristic of a growing system represents a low-input system, which therefore implies a limited capital dedicated to growing tomatoes [27]. One of the peculiarities of the growers in this group is the combination of the varieties of tomatoes in the plots. The simultaneous use of these types of seeds in the system makes it possible to maintain biodiversity and therefore contributes to ecological intensification [48–50]. Indeed, interspecific diversity has already proven itself in agronomy, while intraspecific diversity is about developing new ways of growing crops, including new ideotypes of varieties [49, 51]. The management of biotic and abiotic stresses could be improved by a mixture of varieties with diverse and varied abilities, which will lead to an improvement in yield stability and product quality [48–50, 52]. However, they can solve the availability problem and reduce production costs by using SVs and obtaining seeds from previous seasons [53].

The need to diversify agricultural products is reflected in the association of crops (mainly in the locality of Foumbot) in the third type of cropping system [27]. This association mainly included not only *Solanum nigrum*, but also *Zea mays* and *Phaseolus vulgaris* in a few rare cases. However, in most cases, given the wide range of pests that can cause significant damage to plants in the Solanaceae family, this practice is not conducive to good sanitary management of tomato fields [20, 24]. However, this system has the lowest losses before harvest. Due to the complementary and synergistic effects resulting from the implementation of the modes of action of the different molecules involved, the simultaneous use of contact and systemic fungicides in this group would have a significant advantage [54, 55]. The mixing of varieties is also positioned as an asset in the regulation of losses due to diseases. Indeed, the reduced densities of susceptible plants, coupled with the barrier effects of resistant plants and induced resistance, are considered to represent factors in controlling disease aggressiveness [56–59]. The great diversity of these systems hides solutions that must be understood and refined in order to reduce production losses to a significant level and increase the availability of tomato fruit on the market.

### 4.2. Determinants of Preharvest Tomato Production Losses

Our results show that fertilization is the cropping system component that best explains PHLs observed in tomato growers in the study area. This trend of losses could be justified by the uncontrolled use of synthetic fertilizers combined with insufficient soil fertility management [13–16]. The imbalance between the macro- and microelements in the plant nutrition would favor the fall of the flowers and the fruits, thus causing a part of the losses observed in the preharvest period for most of the tomato growers [13–16]. In fact, over the past few decades, a steady decline in the efficiency of fertilizer use has been reported [12].

Also, our results show that crop association was a determinant of growers' losses during the postharvest period. However, in most cases, this practice is not conducive to a good sanitary management of the tomato plots, considering the wide range of pests and diseases that can cause significant damage to the plants of the nightshade family [20, 24]. Thus, the main causes of fruit drop can be physiological, nutritional, and pathological [12].

While the study highlights fertilization as a key factor influencing PHLs, it may overlook other important contributors such as pest and disease management, irrigation practices, and climatic conditions [12, 24]. A more holistic approach that considers multiple factors could provide a better understanding of the complexities involved in tomato production. Also, the study may not account for long-term trends in fertilizer use and its effects on soil health and crop yields. Without longitudinal data, it is difficult to assess whether observed trends are consistent over time or influenced by short-term factors [60, 61].

To improve tomato production, policymakers should develop agricultural policies that take into account multiple factors that influence production [62, 63]. In addition, there is a need for increased investment in agricultural research that focuses on the long-term effects of fertilizer use, soil health, and integrated pest management strategies [64]. Encouraging and funding longitudinal studies can help track agricultural trends over time [60, 61]. In addition, implementing farmer training programs that emphasize best practices in fertilization, pest management, and irrigation can improve farmers' knowledge and skills, ultimately increasing the efficiency of tomato production [65]. Farmers should also prioritize soil health and implement conservation practices, such as cover crops and crop rotation, to improve the long-term fertility and resilience of their fields [66]. This can result in improved water and nutrient retention, reduced erosion, and overall improved soil health.

### 4.3. Sociodemographic Characteristics of Tomato Growers

Our findings show that farmers' choice of cropping systems is determined by their age, gender, level of education, and whether or not they belong to a FO [67]. Age has an impact on production systems in that older producers may have knowledge and skills acquired through experience, while younger people may be more flexible and willing to adopt new practices [68]. In production and resource management, women and men often have different roles and responsibilities. But in this case, the overwhelming dominance of the male gender in this activity is a reflection of a production that is primarily oriented toward the marketing and the increase of the growers' income [27, 69–71]. People with more education may have access to more modern and efficient information and techniques, which may have an impact on productivity and product quality [27, 33]. Those who have access to land and resources may be more self-sufficient and productive, while those who do not have access may be more dependent on outside labor or resources [27, 33, 68]. Marital status influences producers' behavior in that married people may have family responsibilities and economic obligations that affect their production choices, while single people may have more freedom to explore different production options [27, 68].

### 4.4. Cultivation Technique and Process

Our study results show that tomato growers prefer planting densities between 25,000 and 50,000 plants/ha [72, 73] showed that planting densities above 35,000 plants/ha resulted in better growth, development, and yield performance. Crop combinations such as *Solanum nigrum*, *Zea mays*, and *Phaseolus vulgaris* aim to diversify agricultural production [27], and the strong trend toward ridging aims to improve drainage, aerate the soil, and conserve moisture, all of which promote crop growth [42]. Simultaneous use of standard and hybrid seeds in the system is an atypical practice of these growers. This practice allows for the preservation of biodiversity and thus contributes to ecological sustainability [48, 50, 52].

In addition, our results show that almost all of the tomato growers interviewed opt for organo-mineral fertilization in their technical itineraries. The use of organic and inorganic fertilization in combination is recommended to improve soil fertility, crop yield, and crop production efficiency in Africa south of the Sahara [5]. In fact, it is well established that ferruginous soil, which is mainly found in these areas, is physically and chemically limited in nutrients essential for crop growth and development [36]. The use of synthetic fertilizer, with its ability to release nutrients quickly into the soil at high rates, is therefore justified to a certain extent [39].

Moreover, the use of organic matter makes it possible to improve the physicochemical characteristics of soils with a fairly significant positive impact on sustainable fertility management [43, 47, 74]. The ability to improve soil characteristics becomes a significant asset for producers, in addition to the residual effect of organic amendments favorable to the crops that will succeed the tomato on these plots [43, 46, 47]. Nevertheless, the quantities of organic manure used in this cropping system are still far from the 10 t·ha-1 recommended for tomato production by Adekiya and Agbede [75]. This would be justified by the limited amount of organic manure available, which is unable to satisfy the real mineral needs of plants, the little produced being wasted for lack of minimum technical skills [76, 77].

### 4.5. Diseases and Pesticide Management

Results of our study show that growers prefer to use both systemic and contact fungicides in their plant health management. This is due to complementary and synergistic effects that result from implementing modes of action of different molecules [54, 55]. This has not only reduced the impact of pathologies on the health of the crop but has also promoted better management of the pathologies by reducing their impact on crop performance [54, 55].

Pesticidal plants are rarely used by gardeners despite their known advantages. In fact, some of the reasons that do not encourage their use by growers are that the time required to prepare extracts is often considered too long, the number of treatments required too high, and the specificity of these extracts [2, 78]. As far as effectiveness is concerned, the slow onset of action, low residual activity, and very limited spectrum of activity compared to synthetic products are often considered a disadvantage for farmers [2, 78, 79]. When they are sold commercially, these extracts or formulations are relatively more expensive than synthetic pesticides [80].

### 4.6. Production and Production Losses

Tomato growers in the study area have higher average yields than the national average, primarily due to the use of high inputs, resulting in more productive cropping systems [39]. Similarly, chemical control of pests and diseases by combining molecules is proving to be an important asset in improving production [54, 55]. The existence of FO specialized in vegetable production offers farmers technical and technological assistance that can contribute significantly to the optimization of their cropping systems [33].

Our results show that the tomato growers are experiencing losses in production, even though, according to their perception, these losses are relatively small. Crop diseases and pests are the main culprits [20, 24, 40, 41]. Some diseases affect all plant organs and may cause as much as 100% loss in production before the fruit ripens and is picked [20, 40]. As well as disease, pests may affect the quantity and quality of production by causing damage, particularly to the fruit [20, 40]. Effective control throughout the crop cycle is not guaranteed by using chemical methods to control these pests [40]. In the same way, certain abiotic stresses may also have an impact on the fall of flowers and fruits, which may result in a loss of income for the producers. Some climate hazards (heavy rainfall, hail, heatwaves, etc.) can be linked to crop loss [24].

## 5. Conclusion

For the first time, tomato cropping systems in the western Cameroon region were characterized and the determinants of PHLs were identified. The results showed that there is a diversity that gives rise to the three main systems identified. This diversity is mainly expressed in losses, fertilization, cultivated area, nursery period, and plant density. The prevalent cropping system is a high-input system with relatively high production losses. The characterization of these systems, which implies a poor knowledge of the state of fertility and, as a consequence, inappropriate management of fertilizers, largely justifies the losses observed by the growers; indeed, this study has allowed the association of the use of synthetic fertilizers, such as di-amino phosphate and the complex fertilizer formula 13-13-21 + 3S + 0.01B + 0.01Zn, with the losses observed during the preharvest phase. It should be pointed out, however, that low losses were observed in the case of growers with an associative system of cultivation. The improvement of tomato production in this part of the country must take into account technical factors, especially the physicochemical factors of the soil. This can be done by: (1) conducting comprehensive soil fertility assessments in different tomato production systems to adjust fertilization practices accordingly and optimize fertilizer use; (2) developing and disseminating specific fertilization protocols based on soil analysis and crop needs to minimize nutrient imbalances and reduce PHLs; (3) implementing farmer training programs focusing on best practices in soil management, fertilization, and crop management techniques. This will enable farmers to make informed decisions to improve the efficiency of their tomato production.

## Figures and Tables

**Table 1:** Description of variables used to characterize tomato-based cropping systems and identify determinants of preharvest losses.

No	Variables	Codes	Description
*Sociodemographic*			
1	Gender	GEN	Grower's gender
2	Age	Age	Number of years since birth
3	Level of education	LOE	Highest level of formal education achieved by the grower
4	Origin	Or	Grower is native/non-native
5	Marital status	MS	The producer is single/cohabiting/married

*Agricultural resource factors*			
6	Land acquisition method	LAM	The farmer purchase/leasing/gift/inheritance the cultivated land
7	Tomato area size	AS	Area of land used for tomato production

*Management factors*			
8	Cropping system	IC	The grower opts for monocultures or polycultures when producing tomatoes
9	Crop rotation	CR	The grower rotates or fallows after tomato production
10	Type of seedling	SL/DL	The grower opts for single row or double row sowing
11	Tillage type	Pl	The grower opts for minimal/ridge/localized plowing
12	Member of a farmers' organization	FO	The grower is a member or not of a producer association in his production area
13	Nursery time	NT	Plant life from nursery seed to transplant
14	Plant density	Dty	Number of plants per unit area
15	Types of fertilization	TOF	The grower opts for synthetic/organic and synthetic fertilization
16	Quantity of synthetic fertilizer	QMF	Amount of synthetic fertilizer used for a production cycle
17	Organic fertilizer quantity	QOF	Amount of organic fertilizer used for a production cycle
18	Types of organic fertilizers	BD/LC/PM	The grower opts for the use of broiler droppings/laying chickens/pig manure
19	Use of bioregulators	UBR	The grower may or may not use bioregulators during his production cycle
20	Varieties used	SV/HV	The grower opts for the use of standard/hybrid varieties

*Disease and pesticide management*			
21	Symptoms identification	SID	The grower is able to identify the symptoms in the field
22	Disease identification	DID	The grower is able to identify diseases in the field
23	Types of fungicides	TFG	The grower opts for the use of contact/systemic/systemic and contact fungicides
24	Use of biopesticides	UBP	The grower may or may not use biopesticides during the crop cycle
25	Knowledge of pesticidal plants	KPP	The grower is aware of the existence of pesticidal plants

*Production and production losses*			
26	Preharvest losses	PHL	The producer observes losses during the preharvest phase
27	Harvest losses	HL	The producer observes losses during the harvest
28	Number of harvests	NOH	The number of harvests made by the producer during a production cycle
29	Yield	Yld	The number of crates obtained by the producer during a production cycle
30	Tomato sales point	TSP	Preferential place of sale of the production by the producer

*Geographical factors*		Locality	
31	*Bansoa*	*Bansoa*	The grower's production basin is *Bansoa*
32	*Dschang*	*Dschang*	The grower's production basin is *Dschang*
33	*Foumbot*	*Foumbot*	The grower's production basin is *Foumbot*

**Table 2:** Results of factor analysis for mixed data.

Characteristic	Dim.1	Dim.2	Dim.3	Dim.4	Dim.5	Dim.6	Dim.7
Area (m^2^)	0.46	**0.64**	0.10	0.35	−0.17	0.12	−0.08
Nursery time	−0.29	−0.11	0.26	−0.37	−0.11	**0.62**	−0.19
Plant density	0.53	−0.09	0.12	−0.05	0.33	−0.09	**−0.56**
Yield (number of baskets·ha^−1^)	0.17	**0.71**	0.30	0.49	0.07	0.12	0.01
Loss (number of baskets·ha^−1^)	**−0.64**	−0.45	0.22	0.31	0.06	−0.11	0.02
Organic soil improver (kg·ha^−1^)	**−0.48**	0.47	0.16	0.02	0.33	0.07	0.24
Synthetic fertilizer (kg·ha^−1^)	**−0.56**	−0.29	0.35	0.28	0.24	−0.08	0.32

Eigenvalue	3.70	1.95	1.53	1.29	1.18	0.98	0.93
Percentage of variance	23.18	12.21	9.55	8.05	7.35	6.10	5.83
Percentage of cumulative variance	23.18	35.40	44.95	53.00	60.35	66.45	**72.29**

*Note:* The values in bold are the ones that make the greatest contribution to the construction of the FAMD and CDA axes.

**Table 3:** Typology of tomato cropping systems in the Western Highlands of Cameroon.

Characteristic	Cropping systems		
	Type 1	Type 2	Type 3
Nursery time (day)	26.00 ± 5.00	25.00 ± 7.00	23.00 ± 4.00
Synthetic fertilizer (kg ha^−1^)	1210.00 ± 457.00	722.50 ± 446.40	592.10 ± 378.00
Plant density	34,665.00 ± 9547.00	39,540.20 ± 25,573.90	72,045.30 ± 45,548.90
Organic amendment (kg ha^−1^)	1871.00 ± 906.00	3136.70 ± 2085.40	584.10 ± 523.40
Yield (number of baskets ha^−1^)	669.00 ± 509.00	1587.30 ± 1784.60	936.60 ± 787.20
Area (m^2^)	3597.10 ± 2225.50	8684.40 ± 8089.10	8974.10 ± 8056.00
Preharvest losses (number of baskets ha^−1^)	91.00 ± 49.00	18.00 ± 22.00	7.00 ± 10.00
Percent of preharvest losses	14	1	1

Variety	Hybrid (100%)	Hybrid (100%)	Standard and hybrid (70%)
Seedling line	Simple (97%)	Simple (97%)	Binoculars (66%) simple (34%)
Cultivation system	Pure (93%)	Pure (93%)	Intercropping (59%)
Type of fungicide	Systemic (100%)	Systemic and contact (100%)	Systemic and contact (74%)
Tillage type	Ridge (92%)	Flat (79%)	Ridge (100%)

**Table 4:** Correlation between canonical axes and variables.

Variables	Axis 1	Axis 2
Area size (m^2^)	0.35217980	−0.29622581
Standard varieties	0.02531453	**−0.80373175**
Hybrid varieties	0.03122268	**0.58399190**
Nursery time	−0.09364468	0.24622957
Tillage type	**−0.86119309**	−0.17166147
Plant density	0.03278324	**−0.61656520**
Type of fertilizer	0.02171583	0.40617457
Type of fungicide	0.08797165	0.44172565
Yield (baskets·ha^−1^)	0.38299222	0.06650284
Preharvest losses (number of baskets·ha^−1^)	**−0.59188346**	**0.56284695**
Organic fertilizer (kg·ha^−1^)	0.40244382	**0.50586404**
Synthetic fertilizer (kg·ha^−1^)	−0.34852724	0.40189815
Intercropping	0.16462747	**−0.55772449**

*Note:* The values in bold are the ones that make the greatest contribution to the construction of the FAMD and CDA axes.

**Table 5:** Comparison of qualitative and quantitative variables between tomato cropping systems.

Characteristic	Overall *N* = 127^1^		1 *N* = 66^1^		2 *N* = 34^1^		3 *N* = 27^1^		*p* value
*Qualitative variables*									
Standard varieties	Number of respondents and percentage in brackets								< 0.001⁣^∗∗∗^^3^
No	103	(81)	64	(97)	31	(91)	8	(30)	
Yes	24	(19)	2	(3)	3	(9)	19	(70)	
Hybrid varieties									< 0.001⁣^∗∗∗^^4^
No	8	(6)	0	(0)	0	(0)	8	(30)	
Yes	119	(94)	66	(100)	34	(100)	19	(70)	
Tillage type									< 0.001⁣^∗∗∗^^4^
Flat	27	(21)	0	(0)	27	(79)	0	(0)	
Billon	95	(75)	61	(92)	7	(21)	27	(100)	
Located	5	(4)	5	(8)	0	(0)	0	(0)	
Type of seedling									< 0.001⁣^∗∗∗^^4^
Twin lines	21	(17)	2	(3)	1	(3)	18	(67)	
Single lines	106	(83)	64	(97)	33	(97)	9	(33)	
Intercropping									< 0.001⁣^∗∗∗^^3^
No	99	(78)	62	(94)	26	(76)	11	(41)	
Yes	28	(22)	4	(6)	8	(24)	16	(59)	
Type of fertilization									0.002⁣^∗∗^^4^
Synthetic	4	(3)	0	(0)	0	(0)	4	(15)	
Organic and synthetic	123	(97)	66	(100)	34	(100)	23	(85)	
Type of fungicide									< 0.001⁣^∗∗∗^^4^
Contact	5	(4)	0	(0)	0	(0)	5	(19)	
Systemic	6	(5)	4	(6)	0	(0)	2	(7)	
Contact and systemic	116	(91)	62	(94)	34	(100)	20	(74)	
Quantitative variables	Mean and standard deviation in brackets								
Area size (m^2^)	6102	(6404)	3597	(2243)	8684	(8211)	8974	(8210)	< 0.001⁣^∗∗∗^^2^
Nursery time	24.8	(5.6)	25.8	(5.2)	24.5	(7.0)	22.6	(3.6)	< 0.001⁣^∗∗∗^^2^
Plant density	43,686	(28,266)	34,665	(9620)	38,676	(18,007)	72,045	(46,417)	< 0.001⁣^∗∗∗^^2^
Synthetic fertilizer (kg·ha^−1^)	901	(498)	1120	(460)	723	(453)	592	(386)	< 0.001⁣^∗∗∗^^2^
Organic fertilizer (kg·ha^−1^)	1936	(1562)	1871	(906)	3137	(2117)	584	(533)	< 0.001⁣^∗∗∗^^2^
Yield (number of baskets·ha^−1^)	937	(1121)	669	(513)	1587	(1812)	772	(711)	0.14^2^
Preharvest losses (number of baskets·ha^−1^)	54	(54)	91	(49)	18	(22)	7	(10)	< 0.001⁣^∗∗∗^^2^

^1^Mean (SD); *n* (%).

^2^Kruskal–Wallis rank sum test.

^3^Pearson's chi-squared test.

^4^Fisher's exact test.

⁣^∗∗^very significant.

⁣^∗∗∗^highly significant.

**Table 6:** Sociodemographic characteristics of tomato growers in the west region of Cameroon.

Characteristic	Overall *N* = 196^1^		*Bansoa N* = 69^1^		*Dschang N* = 67^1^		*Foumbot N* = 60^1^		*p* value
Age	Frequency of respondents and percentage in brackets								< 0.001^2^
(15–30)	51	(26)	34	(50)	9	(13)	8	(13)	
(30–45)	78	(40)	21	(31)	32	(48)	25	(42)	
(45–60)	49	(25)	9	(13)	20	(30)	20	(33)	
(60–80)	17	(26)	4	(6)	6	(9)	7	(12)	
Gender									< 0.001^2^
Feminine	19	(10)	1	(1)	14	(21)	4	(7)	
Masculine	177	(90)	68	(99)	53	(79)	56	(93)	
Education									< 0.001^3^
Out of school	6	(3)	2	(3)	0	(0)	4	(7)	
Primary	34	(17)	11	(16)	3	(5)	20	(33)	
Secondary	95	(49)	27	(39)	36	(54)	32	(53)	
Academic	61	(31)	29	(42)	28	(42)	4	(7)	
Origin									0.8^2^
Non-natives	51	(26)	20	(29)	17	(25)	14	(23)	
Natives	145	(74)	49	(71)	50	(75)	46	(77)	
Marital status									< 0.001^3^
Single	59	(30)	38	(55)	12	(18)	9	(15)	
Cohabitation	2	(1)	2	(3)	0	(0)	0	(0)	
Married	135	(69)	29	(42)	55	(82)	51	(85)	
Area (m^2^)									< 0.001^3^
(0–10,000)	173	(89)	58	(84)	67	(100)	48	(81)	
(10,000–20,000)	16	(8)	9	(13)	0	(0)	7	(12)	
(20,000–80,000)	6	(3)	2	(3)	0	(0)	4	(7)	

^1^ *n* (%).

^2^Pearson's chi-squared test.

^3^Fisher's exact test.

**Table 7:** Farming practices of tomato growers in western Cameroon.

Characteristic	Overall *N* = 196^1^		*Bansoa N* = 69^1^		*Dschang N* = 67^1^		*Foumbot N* = 60^1^		*p* value
Plant density per ha	Frequency and percentage of respondents in brackets								
(0–25,000)	35	(18)	16	(24)	11	(16)	8	(14)	
(25,000–50,000)	120	(62)	38	(56)	56	(84)	26	(44)	
(50,000–75,000)	13	(7)	5	(7)	0	(0)	8	(14)	
(75,000–100,000)	14	(7)	7	(10)	0	(0)	7	(12)	
(100,000–200,000)	12	(6)	2	(3)	0	(0)	10	(17)	
Tillage type									< 0.001^2^
Ploughed	40	(20)	29	(42)	2	(3)	9	(15)	
Ridges	149	(76)	35	(51)	65	(97)	49	(82)	
Minimum	7	(4)	5	(7)	0	(0)	2	(3)	
Type of seedlings									< 0.001^3^
Double lines	41	(21)	3	(4)	0	(0)	38	(63)	
Single lines	155	(79)	66	(96)	67	(100)	22	(37)	
Cropping system									< 0.001^3^
Mixed cropping	47	(24)	8	(12)	2	(3)	37	(62)	
Monocropping	149	(76)	61	(88)	65	(97)	23	(38)	
Crop rotation									< 0.001^3^
No	36	(19)	7	()	4	(6)	25	(42)	
Yes	159	(82)	62	()	63	(94)	34	(58)	

^1^ *n* (%).

^2^Pearson's chi-squared test.

^3^Fisher's exact test.

**Table 8:** Fertilization practices of tomato growers in the western part of Cameroon.

Characteristic	Overall *N* = 196^1^		*Bansoa N* = 69^1^		*Dschang N* = 67^1^		*Foumbot N* = 60^1^		*p* value
Type of fertilization	Frequency of respondents and percentage in brackets								0.002^2^
Synthetic	5	(3)	0	(0)	0	(0)	5	(8)	
Organic and synthetic	191	(97)	69	(100)	67	(100)	55	(92)	
Quantity of organic fertilizer (kg)									< 0.001^3^
(0–501)	92	(49)	32	(46)	30	(46)	30	(56)	
(501–1001)	49	(26)	12	(17)	24	(36)	13	(24)	
(1001–2001)	26	(14)	8	(12)	11	(17)	7	(13)	
(2001–20,000)	22	(12)	17	(25)	1	(2)	4	(7)	
Quantity of synthetic fertilizer (kg)									0.079^2^
(0–501)	136	(71)	52	(80)	49	(73)	35	(59)	
(501–1001)	41	(22)	10	(15)	13	(19)	18	(31)	
(1001–2001)	10	(5)	1	(2)	5	(8)	4	(7)	
(2001–4000)	4	(2)	2	(3)	0	(0)	2	(3)	
Use of bioregulators									0.14^3^
No	144	(74)	55	(80)	50	(77)	39	(65)	
Yes	50	(26)	14	(20)	15	(23)	21	(35)	

^1^ *n* (%).

^2^Pearson's chi-squared test.

^3^Fisher's exact test.

**Table 9:** Disease perception and management by tomato growers in western Cameroon.

Characteristic	Overall *N* = 196^1^		*Bansoa N* = 69^1^		*Dschang N* = 67^1^		*Foumbot N* = 60^1^		*p* value
Symptoms identification	Frequency of respondents and percentage in brackets								0.4^2^
No	4	(2)	0	(0)	2	(3)	2	(3)	
Yes	192	(98)	69	(100)	65	(97)	58	(97)	
Diseases identification									< 0.001^3^
No	27	(14)	12	(17)	0	(0)	15	(26)	
Yes	165	(86)	57	(83)	65	(100)	43	(74)	
Types of fungicides									< 0.001^2^
Contact	11	(6)	0	(0)	0	(0)	11	(20)	
Systemic	13	(7)	5	(7)	0	(0)	8	(15)	
Systemic and contact	166	(87)	64	(93)	67	(100)	35	(65)	
Use of biopesticides									0.8^2^
No	185	(95)	66	(96)	64	(96)	55	(93)	
Yes	10	(5)	3	(4)	3	(5)	4	(7)	
Knowledge of pesticidal plants									< 0.001^3^
No	104	(53)	48	(70)	18	(27)	38	(64)	
Yes	91	(47)	21	(30)	49	(73)	21	(36)	

^1^ *n* (%).

^2^Pearson's chi-squared test.

^3^Fisher's exact test.

**Table 10:** Tomato growers' yield and production losses in western Cameroon.

Characteristic	Overall, *N* = 196^1^	*Bansoa*, *N* = 69^1^	*Dschang*, *N* = 67^1^	*Foumbot*, *N* = 60^1^	*p* value
Number of harvests	6.0 (5.0—7.0)	7.0 (6.0—8.0)	5.0 (5.0—5.5)	6.0 (5.0—6.8)	< 0.001^2^
Yield (baskets ha^−1^)	535 (271—1000)	471 (179—1187)	635 (365–881)	600.0 (300—1212)	0.4^2^
Preharvest losses (number of baskets·ha^−1^)	19 (0–207)	14 (0–200)	32 (0–63)	9 (0–207)	< 0.001^2^
Harvest losses (number of baskets·ha^−1^)	36 (0–250)	19 (0–200)	26 (9–52)	66 (0–250)	< 0.001^2^

^1^Mean (min-max); *n* (%).

^2^Pearson's Chi-squared test.

## Data Availability

The datasets generated during and/or analyzed during the current study are available from the corresponding author upon reasonable request.
